# Lymphatic vessel density as a prognostic indicator in Asian NSCLC patients: a meta-analysis

**DOI:** 10.1186/s12890-018-0702-9

**Published:** 2018-08-06

**Authors:** Shuanglan Xu, Jiao Yang, Shuangyan Xu, Yun Zhu, Chunfang Zhang, Liqiong Liu, Hao Liu, Yunlong Dong, Zhaowei Teng, Xiqian Xing

**Affiliations:** 1 First Department of Respiratory Medicine, Yan’an Hospital Affiliated to Kunming Medical University, No. 245, East Renmin Road, Kunming, 650051 Yunnan China; 2 First Department of Respiratory Medicine, The First Affiliated Hospital of Kunming Medical University, Kunming, 650032 Yunnan China; 3 Department of Dermatology, The Second Affiliated Hospital of Kunming Medical University, Kunming, 650032 Yunnan China; 4 The People’s Hospital of Yuxi City, The 6th Affiliated Hospital of Kunming Medical University, Yuxi, 653100 Yunnan China

**Keywords:** NSCLC, Lymphatic vessel density, LVD, Prognostic, Meta-analysis

## Abstract

**Background:**

To determine the association of lymphatic vessel density (LVD) with the prognosis of Asian non-small cell lung cancer (NSCLC) patients via a meta-analysis.

**Methods:**

Eligible studies were selected by searching PubMed and EMBASE from inception to July 25, 2017. The reference lists of the retrieved articles were also consulted. The information was independently screened by two authors. When heterogeneity was significant, a random-effects model was used to determine overall pooled risk estimates.

**Results:**

A total of 15 studies with 1075 patients were finally included in the meta-analysis. LVD was positively associated with the prognosis of NSCLC in the overall analysis (hazard ratio (HR) 1.14, 95% confidence interval (95% CI): 1.02–1.27, *p* = 0.000, I^2^ = 73.2%). Subgroup analyses were performed on 5 VEGFR-3 groups (*p* = 0.709, I^2^ = 0.0%), 3 LYVE-1 groups (*p* = 0.01, I^2^ = 86.4%), 5 D2–40 groups (*p* = 0.019, I^2^ = 66.2%), and 2 podoplanin groups (*p* = 0.094, I^2^ = 64.5%). Sensitivity analysis indicated robust results. There was no publication bias.

**Conclusions:**

LVD is an indicator of poor prognosis in Asian NSCLC patients.

## Background

Lung cancer is a malignant disease associated with the highest mortality rate (18.2%) among all types of cancer worldwide [1, 2]. Non-small cell lung cancer (NSCLC) represents the majority (~ 85%) of all lung cancer cases, with lung adenocarcinoma (ADC) and squamous cell carcinoma (SCC) being the most frequently diagnosed histological types [3]. Approximately half of all NSCLC patients have metastasis, and this type of cancer is usually diagnosed at advanced stages. Despite great progress in treatment modalities (such as surgical resection, chemotherapy, radiotherapy, targeted therapy, biotherapy, and cellular immunotherapy), the prognosis of NSCLC remains poor, and the long-term survival of NSCLC patients is still dismal [4]. Thus, it is important to find novel prognostic therapeutic targets and precise prognostic markers for this type of cancer.

Cancer relapse and metastasis lead to poor prognosis. The most common mode of metastasis is lymph node metastasis. During the early stages of tumor dissemination, malignant cells spread from primary sites to regional lymph nodes. Therefore, the lymphatic system plays an important role in cancer biology [5]. The formation of new lymphatic vessels (lymphangiogenesis) occurs through several steps, including the migration, proliferation and sprouting of lymphatic endothelial cells, which are triggered by vascular endothelial growth factor receptor (VEGFR)-3, VEGF-C or VEGF-D [6]. The lymphatic vessel density (LVD) is the parameter that is most frequently used to quantify tumor lymphangiogenesis, especially for melanoma [7], oral squamous cell carcinoma [8], thyroid carcinoma [9], colorectal cancer [10], breast cancer [11], and lung cancer [12].

Previous studies have identified novel molecular markers of the lymphatic endothelium that have been used to study tumor-associated lymphangiogenesis via immunochemistry. These markers include VEGFR-3, Lymphatic vessel endothelial hyaluronan receptor-1 (LYVE-1), D2–40, podoplanin, Prox-1 and desmoplakin, among others [13–16]. VEGFR-3, also known as Flt4, is a member of the fms-like tyrosine kinase family, and it specifically binds VEGF-C and VEGF-D. LYVE-1 is a homolog of the vascular endothelium-specific hyaluronan receptor CD44 [17]. The antibody against D2–40 has been shown to specifically recognize the M2A antigen and podoplanin [18, 19]. Podoplanin is a glomerular podocyte membrane mucoprotein [20]. The transcription factor prox-1 is a homolog of the *drosophila* homeobox gene product that is involved in the regulation of early lymphatic development [21]. Desmoplakin, also known as desmosome-related transmembrane protein, is a desmosomal protein expressed at intercellular junctions. Some studies have shown that lymphatic endothelium markers can be used to predict poor prognoses in NSCLC patients [22–24], but other studies have refuted this view [25–27]. Therefore, whether LVD is a prognostic biomarker for the survival of NSCLC patients remains controversial. The aim of this meta-analysis was to examine whether LVD can predict the prognosis of Asian NSCLC patients.

## Methods

### Search strategy

PubMed and EMBASE were searched from inception to July 25, 2017, to find related studies. The search terms used were 1) “Non-small cell lung cancer”, “Non-small cell lung carcinoma”, “NSCLC”, “lung adenocarcinoma”, “adenocarcinoma of lung”, “lung squamous cell cancer”, “squamous cell cancer of the lung”, “lung squamous cell carcinoma”, “squamous cell carcinoma of the lung”, “lung large cell cancer”, “large cell cancer of the lung”, “lung large cell carcinoma”, and “lung large cell carcinoma”; 2) “Lymphangiogenesis”, “Lymphangiogeneses”, “Lymphatic microvessel density”, “Lymphatic vessel density”, “Lymphatic microvessel”, and “Lymphatic vessel”; and 3) “prognostic”, “prognosis”, and “survival”.

#### Study selection

The inclusion criteria were as follows: 1) a cohort study; 2) an Asian study population; 3) diagnosis of NSCLC based on lung histology, with the most important histological types being ADC, SCC and large cell cancer (LCC); 4) evaluation of the association between LVD and the prognosis of NSCLC patients; 5) analysis of lymph microvessel markers by immunohistochemistry; and 6) the presence of sufficient data to calculate the adjusted hazard ratio (HR) or risk ratio (RR) and the corresponding 95% confidence intervals (CIs). Studies were excluded if they had non-human study subjects. If the data were duplicated or the same population was used in more than one study, we chose the most recent or complete study.

#### Data extraction

The eligible studies selected for our meta-analysis were independently evaluated by two reviewers (XXQ and XSL) based on the aforementioned selection criteria. The following information was extracted from the eligible studies: the name of the first author, publication year, study period, country, sample number, sex of patients, median follow-up period, mean age or age range of patients, histology, histological type, TNM stage, and lymphatic endothelium markers (in Table 1). In addition, HR and 95% CIs were evaluated. Two authors (TZW and XSY) summarized the extracted data. Any disagreements were resolved by discussion.

**Table 1:** Characteristics of the 15 studies

Author-year (study period) Country	Sample number	Sex		Median follow-up period (months)	Age: mean age or range	Histology	Histological type				TNM stage	Lymphatic endothelium markers
		Males	Females				ADC	SCC	LCC	Others		
Kitano-2017 (1988–2010) Japan [33]	89	64	25	range 10–153	< 60 y 25 ≥60 y 64	NSCLC	53	36	0	0	II 40 III + IV 49	VEGFR-3
Nunomiya-2014 (2008–2011) Japan [25]	58	50	8	ND	71.3 y	NSCLC 40 SCLC 14 Others 4	ND	ND	ND	ND	I + II 20 III + IV 37	LYVE-1
Hao-2014 (2004–2012) China [34]	140	72	68	ND	≤65 y 56 > 65 y 84	NSCLC	36	39	28	4	I–IIIA	LYVE-1
Zhang-2012 (2003–2006) China [22]	65	38	27	ND	51.5 y (range 32–76 y) < 55 y 26 ≥55 y 39	ADC	65	0	0	0	I + II 38 III + IV 27	D2–40
Dai-2011 (1999–2003) China [35]	98	ND	ND	37.53 ± 4.05	ND	NSCLC	59	39	0	0	ND	Podoplanin
Yamashita-2010 (1993–2000) Japan [36]	117	77	40	68.7	67.8 y (range 47–85 y) < 68 y 67 ≥68 y 50	Stage I NSCLC	78	31	6	2	IA 58 IB 59	VEGFR-3
Chen-2010 (1999–2001) China [37]	52	41	11	ND	51.9 y (range 29–77 y) < 60 y 31 ≥60 y 21	NSCLC	16	23	0	13	I + II 33 III 19	LYVE-1
Sun-2009 (1995–2004) China [38]	82	63	19	ND	< 55 y 40 ≥55 y 42	NSCLC	41	31	10	0	I + II 48 III + IV 34	D2–40
Iwakiri-2009 (1998–1990) Japan [39]	215	159	56	ND	63.0 y (range 53–71.8 y) < 63 y 109 ≥63 y 106	NSCLC	116	82	10	7	I + II 147 IIIA 68	D2–40
Kitano-2009 (ND) Japan [23]	82	45	37	ND	65 y	ADC	82	0	0	0	I + II 65 III + IV 17	Podoplanin
Kadota-2008 (1998–2002) Japan [24]	147	100	47	ND	67 y (range 35–82 y)	NSCLC	93	49	5	0	I + II 108 III 39	D2–40
Ohta-2006 (1981–2004) Japan [40]	44	23	21	20	64.4 y	NSCLC	25	17	0	2	IIIA 35 IIIB 9	D2–40
Kojima-2005 (1981–1998) Japan [26]	129	62	67	69.9	61 y (range 38–78 y)	ADC	129	0	0	0	ND	VEGFR-3
Chen-2004 (1985–1990) Japan [41]	206	148	58	ND	< 64 y 101 ≥64 y 105	NSCLC	116	75	10	5	I + II 144 IIIA 62	VEGFR-3
Arinaga-2003 (1990–1996) Japan [27]	180	133	47	54.6	65 y (range 35–84 y)	NSCLC	65	101	0	14	I + II 130 III 41	VEGFR-3

*NSCLC* non-small cell lung carcinoma, *SCLC* small cell lung carcinoma, *ADC* adenocarcinoma, *SCC* squamous cell cancer, *LCC* large cell cancer, *VEGFR-3* vascular endothelial growth factor receptor-3, *LYVE-1* lymphatic vessel endothelial receptor 1, *y* year, *ND* no data

### Statistical analyses

To compute a pooled HR with a 95% CI, the Q-test and the I^2^ test were used to assess heterogeneity among the studies [28]. We also calculated *P* values for the Q-test, which represented heterogeneity; heterogeneity was present if the P value was less than 0.10. The random-effects model was applied when I^2^ > 50% [29]; otherwise, the fixed-effects model was applied [30]. Subgroup analyses based on lymphatic endothelium markers were performed to further explore the source of heterogeneity. Additionally, Begg’s rank correlation test and Egger’s linear regression test were conducted to assess the extent of potential publication bias [31]. Finally, a sensitivity analysis was performed by sequentially omitting one study per cycle to evaluate the stability of the results [32]. The data analyses were conducted using the STATA statistical software version 12.0 (STATA Corp. LLC, College Station, TX, USA).

## Results

### Literature search and study characteristics

**Fig. 1:**
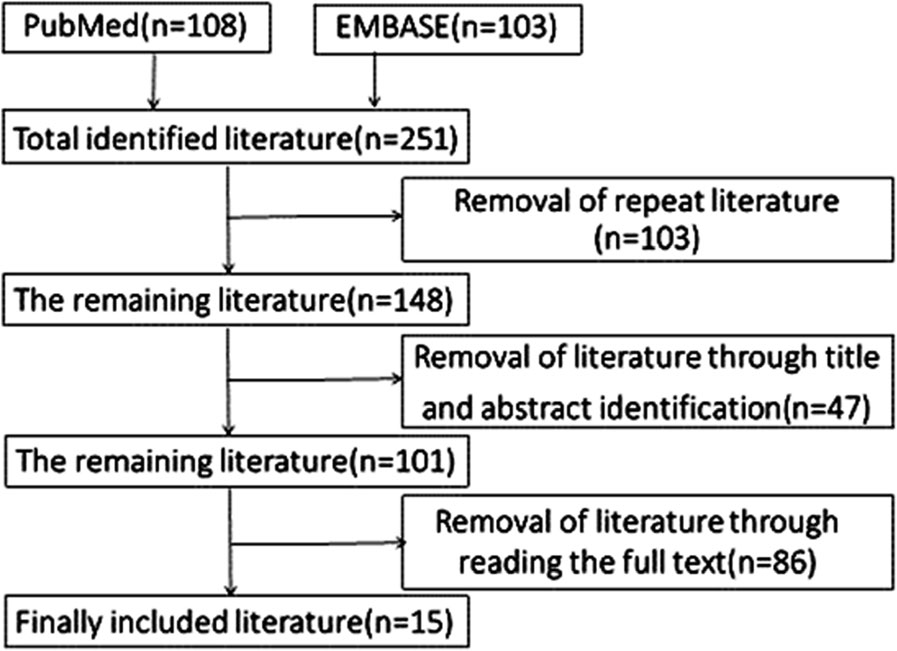
Flow chart of the meta-analysis

Using the predefined search strategy and inclusion criteria, 15 studies [22–27, 33–41] involving 1075 participants were ultimately included in this meta-analysis. The detailed study selection process is presented in Fig. 1. In total, 251 articles (108 from PubMed and 143 from EMBASE) were retrieved. Among these articles, 236 articles were excluded after eliminating duplicates, screening the titles and abstracts, and reviewing the full text. Finally, 15 articles were included in our analysis.

The characteristics of the 15 eligible studies are shown in Table 1. These studies included 1075 participants from Asia, including Japan and China;a total of 11 studies investigated NSCLC, 3 studies investigated ADC, and 1 study investigated lung cancer. All studies used immunohistochemistry to assess LVD using different lymphatic endothelium markers, including VEGFR-3 in 5 studies, LYVE-1 in 3 studies, D2–40 in 5 studies, podoplanin in 2 studies.

### Main analysis

**Fig. 2:**
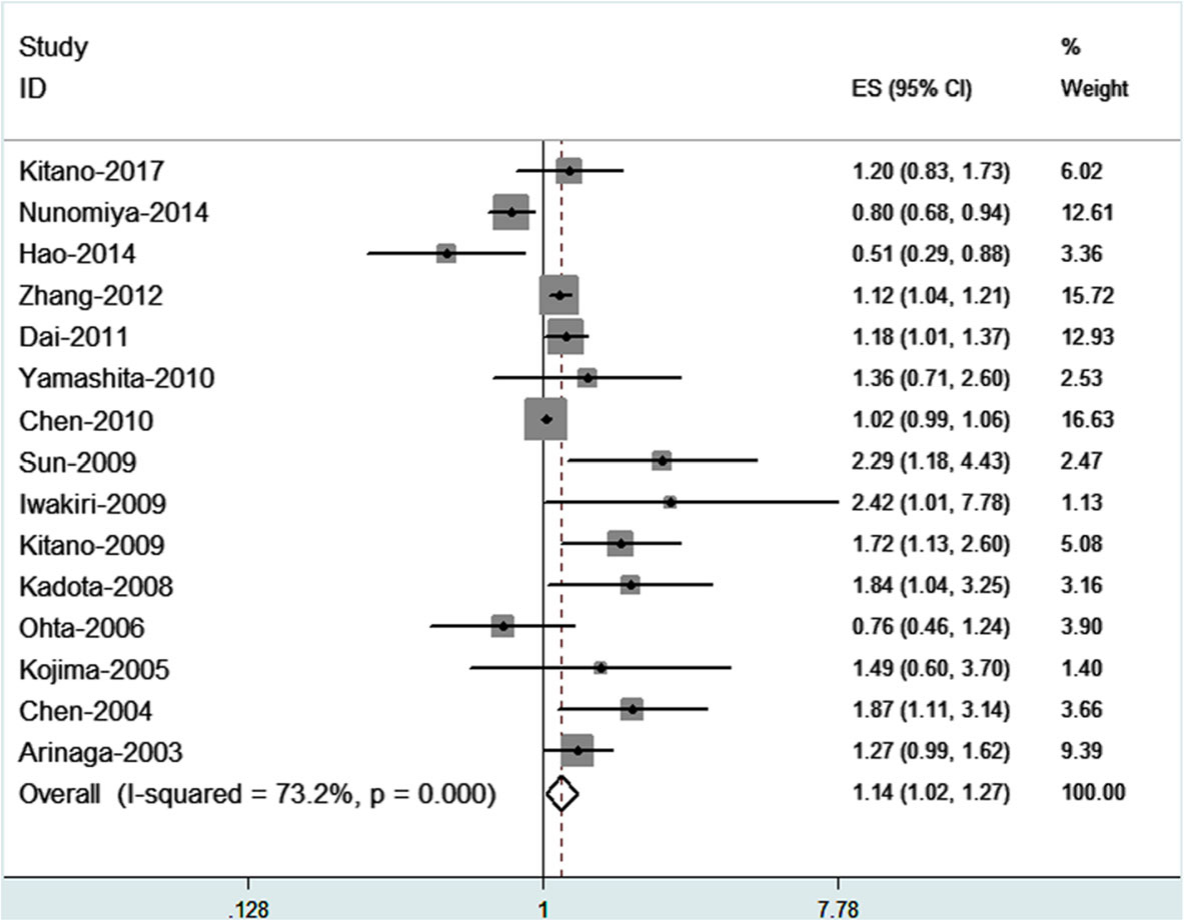
Forest plot of LVD associated with NSCLC prognosis

LVD was positively associated with the prognosis of NSCLC in the overall analysis (HR 1.14, 95% CI: 1.02–1.27) (Fig. 2). However, significant heterogeneity was detected across studies (I^2^ = 73.2%; *P* = 0.000).

### Subgroup meta-analysis

**Fig.3:**
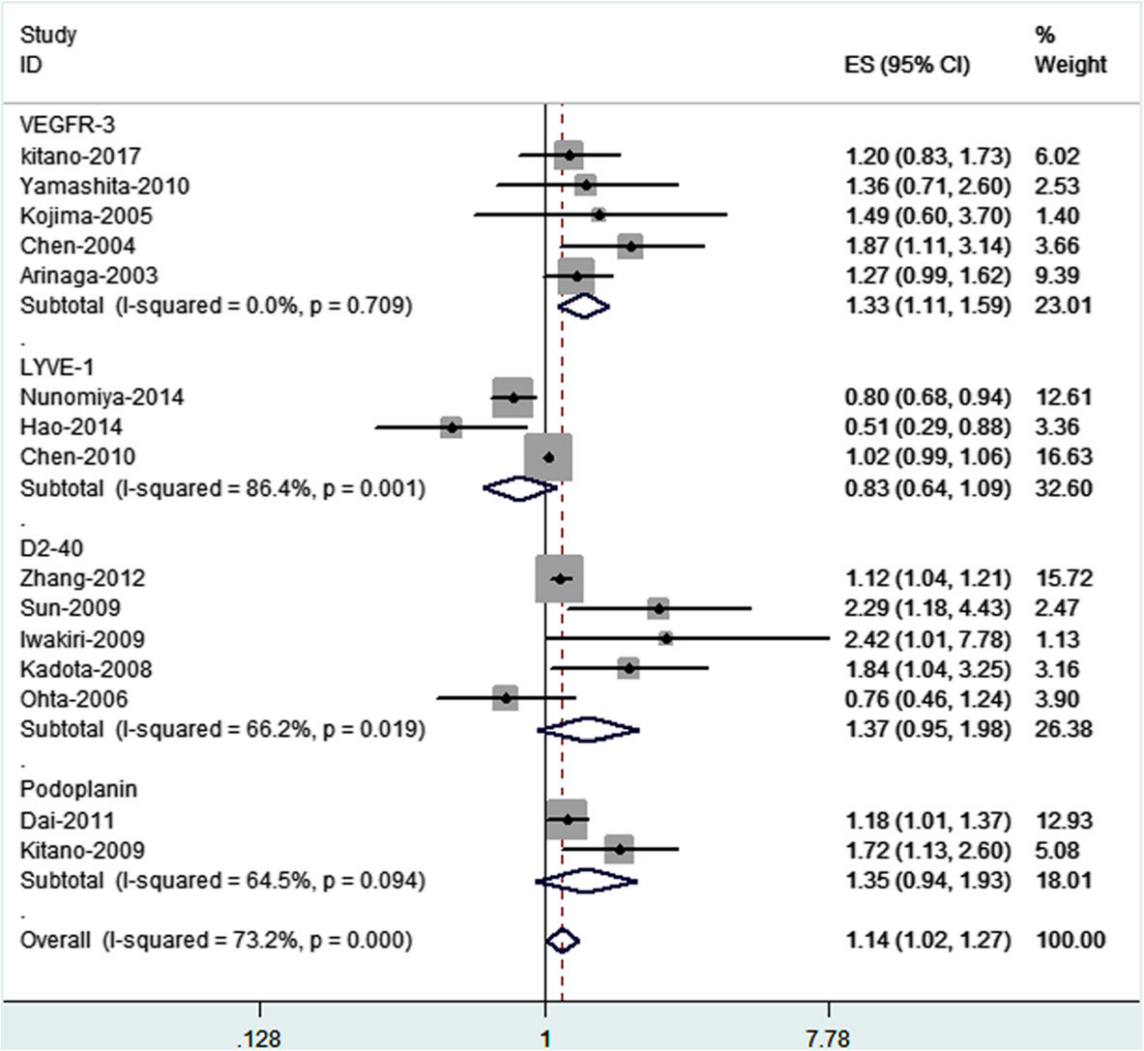
Subgroup analysis of LVD associated with NSCLC prognosis

The results of subgroup analyses using the lymphatic endothelium markers that were selected to evaluate LVD via immunohistochemistry support our findings. A positive relationship was observed between the expression of lymphatic endothelium markers and the prognoses of NSCLC patients (*p* = 0.000, I^2^ = 73.2%). No statistically significant heterogeneity was observed in the 5 VEGFR-3 group (*p* = 0.709, I^2^ = 0.0%); however, there was considerable heterogeneity in the 3 LYVE-1 groups (*p* = 0.01, I^2^ = 86.4%), the 5 D2–40 groups (*p* = 0.019, I^2^ = 66.2%) and the 2 podoplanin groups (*p* = 0.094, I^2^ = 64.5%) (Fig. 3). Nevertheless, the data were not sufficient to determine the prognostic value of LVD among Asian populations based on sex, median follow-up period, mean age or age range, histological type, or TNM stage.

### Sensitivity analysis

**Fig. 4:**
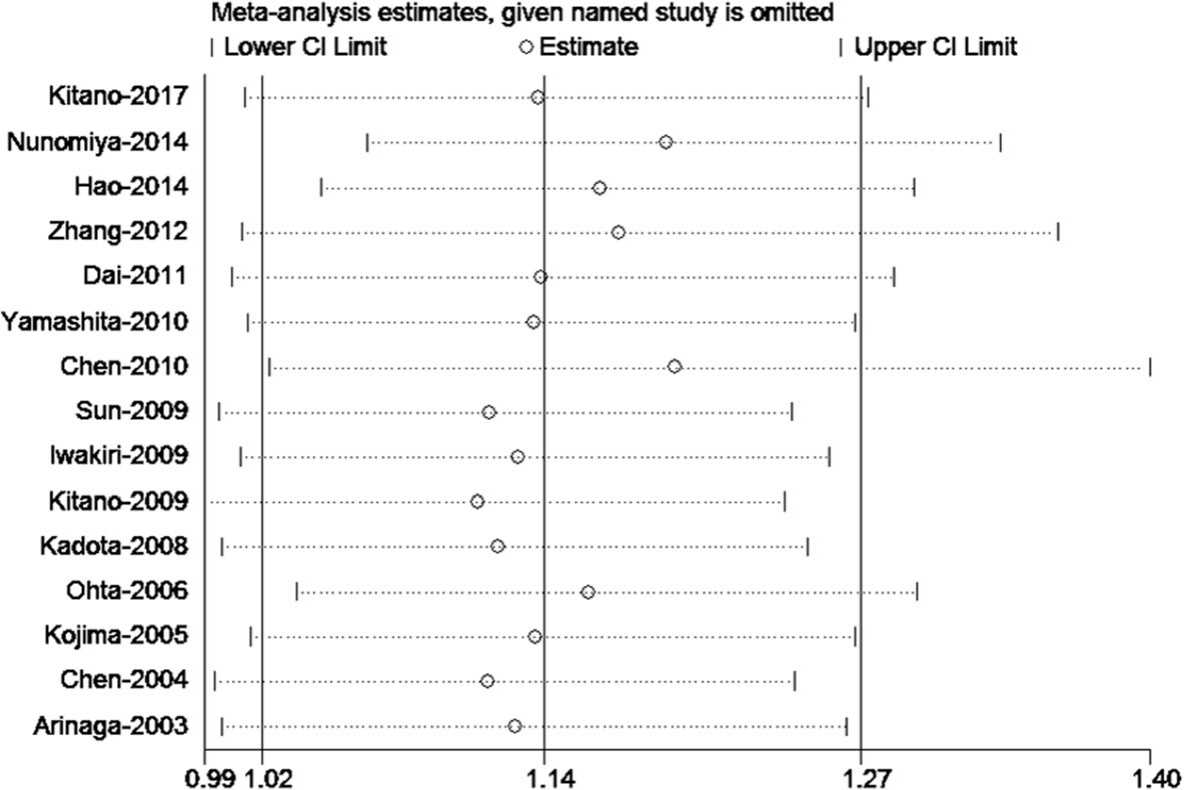
Sensitivity analysis of LVD associated with NSCLC prognosis

To evaluate the robustness of our analysis, we conducted a sensitivity analysis by recalculating the pooled results from the primary analyses after excluding one study per iteration. None of the studies when excluded altered the overall combined results (Fig. 4).

### Publication bias

**Fig. 5:**
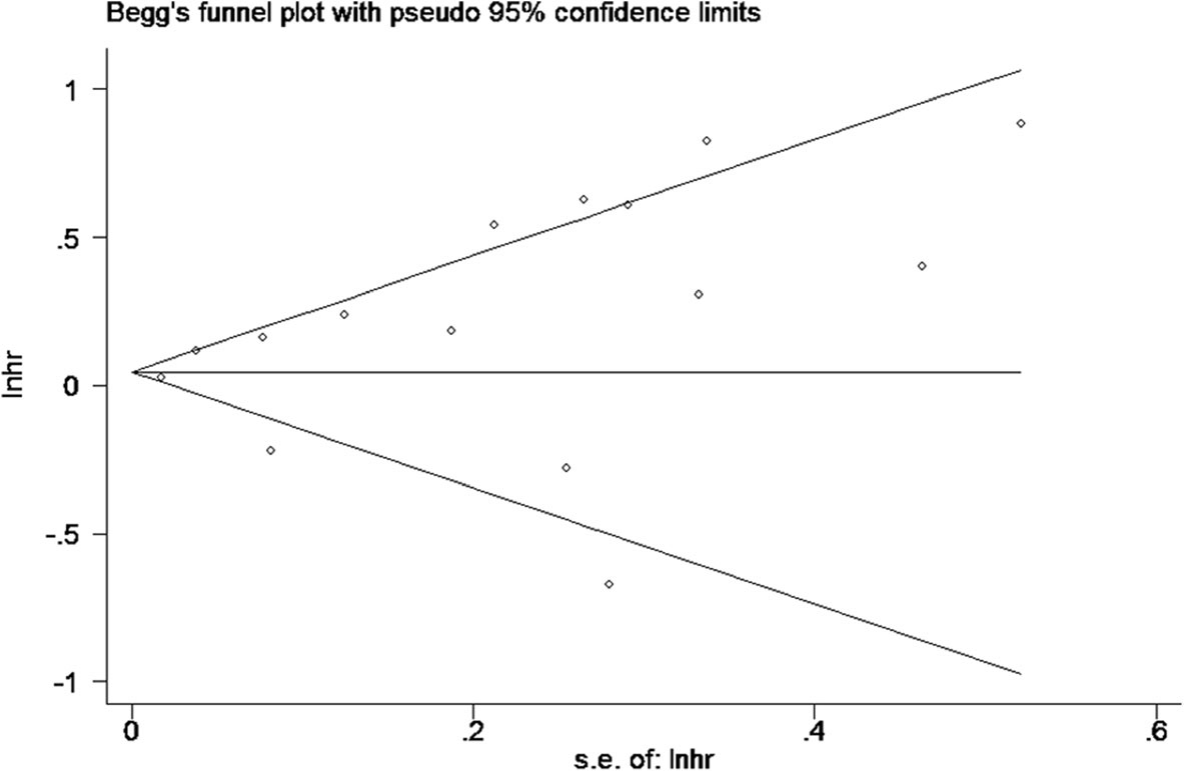
Begg’s funnel plot

**Fig. 6:**
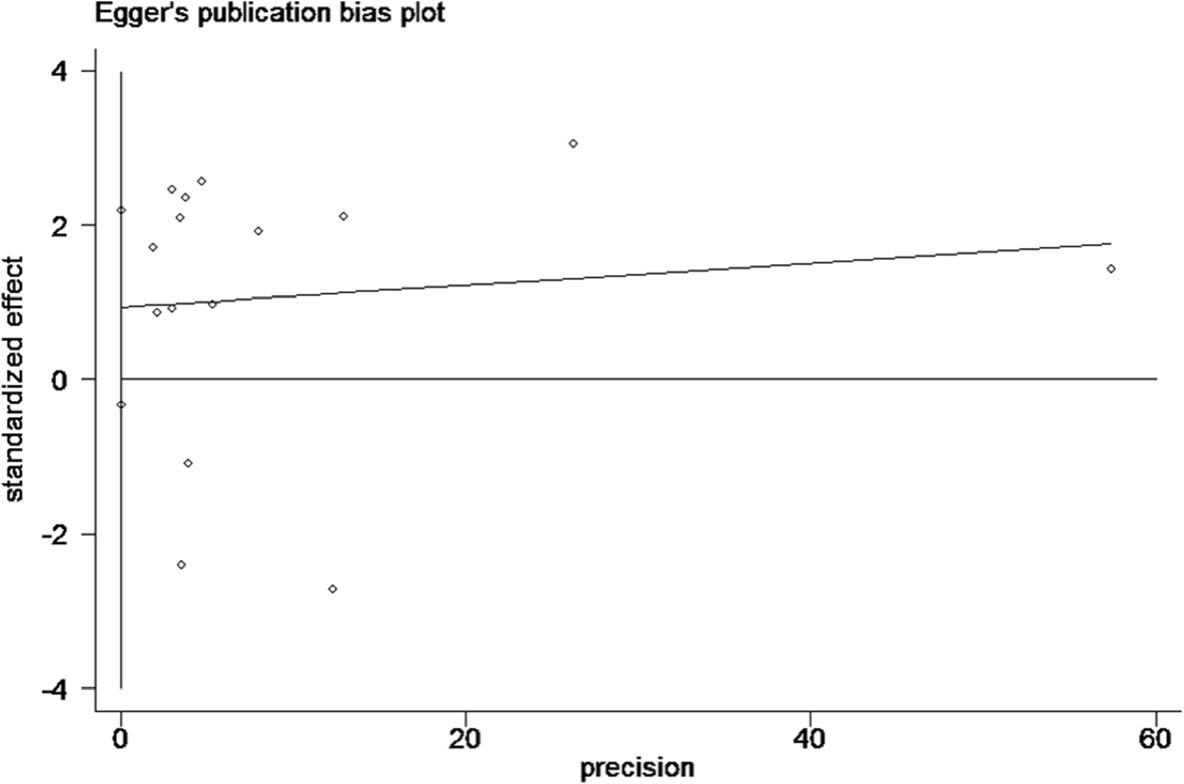
Egger’s publication bias plot

No evidence of publication bias was found based on the Begg’s rank correlation test (*p* > |z| = 0.488) or Egger’s linear regression test (*p* > |z| = 0.133) (Figs. 5 and 6).

## Discussion

NSCLC is the most common subtype of lung cancer, with a high incidence, high mortality, low survival rate, low diagnosis rate and treatment rate. It has been challenging to improve survival rates due to the lack of precise prognostic markers. To overcome this problem, a comprehensive understanding of lymphatic endothelium markers is needed. It is important to examine whether LVD can be an indicator of the prognosis in Asian NSCLC patients.

In our present meta-analysis, LVD was positively associated with the prognosis of NSCLC (HR: 1.14, 95% CI: 1.02–1.27), indicating that high LVD indeed predicts poor survival in Asian NSCLC populations. To date, only Wang and colleagues [42] have described the relationship between LVD and the prognoses of NSCLC patients worldwide. Nevertheless, there was considerable heterogeneity among the included studies, which may make the results unreliable. However, sensitivity analysis did not reveal the source of heterogeneity. Furthermore, subgroup analyses were conducted using lymphatic endothelium markers. Additionally, publication bias was detected. Our study only focused on Asian patients, and thus our results are applicable for Asian populations. Although heterogeneity was also observed, the findings were stable and robust based on our sensitivity analysis. In addition, subgroup analyses were performed based on the four lymphatic endothelium markers to further explore the origin of heterogeneity. Except VEGFR-3, the other three markers gave rise to considerable heterogeneity. Our meta-analysis included five additional studies that were more recent than those included in the study by Wang and colleagues. Moreover, no publication bias was observed in our study. The study by Zheng and colleagues [43] showed that the VEGF family is important for tumorigenesis and metastasis and that high VEGF and/or VEGFR expression, especially VEGF-C/VEGFR-3 co-expression, is indicative of poor survival in patients with NSCLC. However, that study did not evaluate other lymphatic endothelium markers, which were included in subgroup analyses in our study.

The role of LVD as a prognostic predictor in NSCLC remains controversial. Kajita and colleagues were the first to report VEGFR-3 expression in lung cancer cells, but they did not evaluate its impact on the prognosis or the correlation of VEGFR-3 expression with clinicopathologic features in patients with NSCLC [44]. Later, many studies demonstrated that VEGF-C, VEGF-D, VEGFR-3 and other markers are independent markers of poor prognostic in patients with NSCLC. Thus, these markers may be ideal targets for diagnosis or therapy to improve the prognosis of NSCLC patients [34]. The study by Arinaga demonstrated that the combined expression of VEGF-C and VEGFR-3 has a negative impact on the prognosis of patients with NSCLC [27]. In addition, the study by Zhang and colleagues revealed that D2–40-positive peritumoral LVD may be an independent prognostic factor for lung adenocarcinoma. Thus, D2–40-positivity may be used to predict patient prognosis in lung adenocarcinoma. Moreover, the reduction of peritumoral lymphangiogenesis has been suggested to inhibit the metastasis of lung adenocarcinoma [22]. However, some studies have claimed that high LVD may be a marker for good prognosis. The study by Nunomiya and colleagues showed that lung cancer patients with lower LYVE-1 levels have poorer prognoses than patients with higher LYVE-1 levels [25]. Yang and his team demonstrated the role of the epigenetic regulation of desmoplakin in increasing the sensitivity of cancer cells to anticancer drug-induced apoptosis, implying the clinical value of desmoplakin for the treatment of patients with lung cancer [45]. Nevertheless, more studies are needed in the near future to verify whether LVD is indicative of good or bad prognosis in NSCLC patients.

VEGFR-3, D2–40, LYVE-1 and podoplanin are widely used and extremely valuable markers of lymphatic vessels. However, one study has reported that lymphatic endothelium markers are not only expressed on lymphatic vessels but also expressed on blood vessels, tumor cells or in normal tissues [13]. One of the major drawbacks is the lack of specific markers for the lymphatic endothelium. One study [46] indicated that LYVE-1 and Prox-1 are molecular markers of lymphangiogenesis in NSCLC and that they can be used as important markers for the evaluation of lymphatic metastasis and prognoses in patients with NSCLC. Another study [43] showed that high VEGF and/or VEGFR expression is indicative of poor survival in patients with NSCLC and that VEGF-C/VEGFR-3 co-expression is a better prognostic indicator than other markers. Therefore, the evaluation of co-expressed markers may be useful to determine LVD.

Irrespective of its strengths, the meta-analysis also has certain limitations. First, although we searched all retrospective studies for the association between LVD and the prognosis of NSCLC, the eligible studies were restricted to those published in English or Chinese. Because of linguistic barrier, some non-English or non-Chinese studies were excluded. In addition, we also missed some studies that may have been published in books or journals that were not available in the online databases. Additionally, studies with negative data may not have been submitted by investigators, or studies with nonsignificant results may have been rejected by journals. Nevertheless, there was no significant publication bias in our study, although we could not completely rule out publication bias. Second, few studies did not present clear or complete data, making data analysis difficult. When we could not obtain original data from the authors via email or other means, we had to exclude those studies. Third, because of the small number of eligible articles, our study was not the most comprehensive. Fourth, our results cannot be generalized to populations worldwide, especially non-Asian populations. Thus, more comprehensive and higher quality analyses are still required in the future.

## Conclusions

In summary, this meta-analysis indicated that LVD is an indicator of the prognosis of Asian NSCLC patients. However, higher quality and more comprehensive analyses are still needed as more data are published in the future.

## Abbreviations

95% CI: 95% confidence interval
ADC: Adenocarcinoma
HR: Hazard ratio
LCC: Large cell cancer
LVD: Lymphatic vessel density
LYVE-1: Lymphatic vessel endothelial hyaluronan receptor-1
NSCLC: Non-small cell lung cancer
SCC: Squamous cell carcinoma
VEGFR: Vascular endothelial growth factor receptor
